# Provably-Secure (Chinese Government) SM2 and Simplified SM2 Key Exchange Protocols

**DOI:** 10.1155/2014/825984

**Published:** 2014-09-02

**Authors:** Ang Yang, Junghyun Nam, Moonseong Kim, Kim-Kwang Raymond Choo

**Affiliations:** ^1^Information Assurance Research Group, Advanced Computing Research Centre, University of South Australia, Mawson Lakes, SA 5095, Australia; ^2^Department of Computer Engineering, Konkuk University, 268 Chungwondaero, Chungju, Chungcheongbuk-do 380-701, Republic of Korea; ^3^Information Management Division, Korean Intellectual Property Office, 189 Cheongsaro, Daejeon 302-701, Republic of Korea

## Abstract

We revisit the SM2 protocol, which is widely used in Chinese commercial applications and by Chinese government agencies. Although it is by now standard practice for protocol designers to provide security proofs in widely accepted security models in order to assure protocol implementers of their security properties, the SM2 protocol does not have a proof of security. In this paper, we prove the security of the SM2 protocol in the widely accepted indistinguishability-based Bellare-Rogaway model under the elliptic curve discrete logarithm problem (ECDLP) assumption. We also present a simplified and more efficient version of the SM2 protocol with an accompanying security proof.

## 1. Introduction

Due to the potential of elliptic curve cryptography (ECC) to offer similar security to established public-key cryptosystems at reduced key sizes, it has become a subject of research focus. For example, we observe an emerging trend in the use of identity-based (ID-based) cryptography, such as ID-based key agreement protocols using pairings. The latter include ID-based authenticated key agreement (ID-AKA) protocol. ID-AKA protocols (as well as other key establishment protocols such as [1–4]) allow a shared secret key to be established between two or more parties for subsequent cryptographic use. The first two-party ID-AKA protocol was proposed by Shamir, which is based on Weil Pairing [5]. Shamir's protocol requires a trusted key generation center (KGC). Challenges associated with KGC are well documented, and Alriyami and Paterson proposed the first certificateless two-party authenticated key agreement (CTAKA) protocol that does not require a KGC [6]. Since then, a number of CTAKA protocols have been proposed in the literature [7–9]. Most of these CTAKA protocols are, however, based on bilinear pairings. The latter is expensive, especially in comparison to RSA algorithm [10, 11].

A number of recently published certificateless ECC-based AKA protocols that do not require the use of pairings have been proposed. For example, in 2007, Zhu et al. proposed a pairing-free ID-AKA protocol [12]. However, the combination of a pairing-free ID-based signature scheme with the Diffie-Hellman key exchange in the proposed protocol results in larger computation complexity and message size. In addition, the protocol and the ECC-based pairing-free ID-AKA protocol of Cao et al. [13] require three rounds of message exchanges. Another later protocol of Cao et al. reduces the minimum message exchange rounds to two and the protocol was proven secure in the Bellare-Rogaway model [10]. He et al. also independently proposed a two-round certificateless ID-AKA protocol without the use of pairings [14] and a three-round certificateless ID-AKA protocol without the use of pairings [2], respectively.

In 2011, the Chinese government published an ECC-based key exchange protocol, SM2 [15]. According to the official report from the Chinese Government State Cryptography Administration and various media releases, SM2 protocol is mandatory in various cryptographic applications used by Chinese government agencies from 1st July, 2011 [16–18]. A 2005 survey by Boyd and Choo revealed that the purported security of several published ID-based protocols is based on heurstic security arguments. A number of protocols were also found to be proven secure in a restricted model. This study highlighted the need for more rigorously tested identity-based protocols [19]. Surprisingly, we observed that despite the wide usage of the SM2 protocol among Chinese commercial applications/electronics, it does not have a security proof.

A protocol's goal is defined as the properties that the protocol aims to achieve. As Boyd and Mathuria suggested, any attack on a protocol is only valid if it violates some property that the protocol was intended to achieve [20]. Without identifying at an early stage the properties and/or goals that a protocol offers, one can debate the validity of attacks against a published protocol since it may not be clear whether the protocol is not intended to provide assurances against the properties being exploited [21]. This reinforced the importance of having a security proof for protocols, particularly those that are widely used by government agencies and in the private sector.

Our contributions in this paper are two-fold.

1. We prove the SM2 protocol secure in the widely accepted indistinguishability-based model of Bellare and Rogaway under the ECDLP assumption.
2. We propose a simplified version of SM2 protocol that is more efficient, and prove it secure in the Bellare-Rogaway model under the ECDLP assumption.

In the next section, we will briefly review the model that we work in. We revisit the SM2 protocol and prove it secure in Section 3.  Section 4 describes our simplified SM2 protocol and its proof of security. Finally, the last section concludes the paper.

## 2. Overview of the Bellare-Rogaway Model

In the Bellare-Rogaway model [22, 23], the adversary (denoted by *A*) controls the communication channel by interacting with a set of Π_*U*_1_,*U*_2__^*i*^ oracles. Π_*U*_1_,*U*_2__^*i*^ is defined to be the *i*th instantiation of a protocol participant, *U*_1_ in a specific protocol run and *U*_2_ is the other protocol participant, with whom *U*_1_ wishes to establish a secret key. The predefined oracle queries are described informally as follows.

- The Send  (*U*_1_, *U*_2_, *i*, *m*) query allows *A* to send a message *m* to another protocol participant at will. In other words,
  - Π_*U*_1_,*U*_2__^*i*^, upon receiving the query, will compute what the protocol specification demands. The response message and/or decision will then be sent to *A*,
  - if Π_*U*_1_,*U*_2__^*i*^ has either accepted with some session key or terminated, this will be made known to *A*.
- The Reveal  (*U*, *i*) query allows *A* to expose a previously accepted session key. In other words, *U* ^*i*^, upon receiving this query and if it has accepted and holds some session key, will send this session key back to *A*.
- The Corrupt  (*U*) query allows *A* to learn the complete internal state of *U*. This models the real world scenario of a corrupted insider.
- The Test  (*U*_1_, *U*_2_, *i*) query is the only oracle query that does not correspond to any of *A*'s abilities. If Π_*U*_1_,*U*_2__^*i*^ has accepted with some session key and is being asked a Test  (*U*_1_, *U*_2_, *i*) query; then depending on a randomly chosen bit *b*, *A* is given either the actual session key or a session key drawn randomly from the session key distribution.

**Definition 1 (Definition of Partnership).**

Let us denote Π_*A*,*B*_^*i*^ and Π_*B*,*A*_^*j*^ as two oracles in the protocol run. These two oracles are considered partners if and only if

- both Π_*A*,*B*_^*i*^ and Π_*B*,*A*_^*j*^ have accepted the same session key,
- only Π_*A*,*B*_^*i*^ and Π_*B*,*A*_^*j*^ (i.e., no other oracle) have accepted with the same session ID (i.e., SID, which is defined to be the concatenation of the message flows) and agreed on the same set of principals (i.e., the initiator and the responder of the protocol).

**Definition 2 (Definition of Freshness).**

Oracle Π_*A*,*B*_^*i*^ holds a fresh session key at the end of execution, if and only if all the following conditions are satisfied:

- Π_*A*,*B*_^*i*^ has accepted with, or without, a partner oracle Π_*B*,*A*_^*j*^,
- Π_*A*,*B*_^*i*^ and Π_*B*,*A*_^*j*^ (if such a partner oracle exists) has/have not been sent a  Reveal  query,
- both *A* and *B* (if such a partner exists) has/have not been sent a  Corrupt  query.

The definition of security depends on the notions of partnership as outlined in Definition 1 and freshness as outlined in Definition 2 and is defined using the game *G* and played between *A* and a collection of Π_*U*_*x*_,*U*_*y*__^*i*^ oracles for players *U*_*x*_, *U*_*y*_ ∈ {*U*_1_,…, *U*_*N*_*p*__} and instances *i* ∈ {1,…, *N*_*s*_}. *A* runs the game simulation *G*, whose setting is as follows.

- Send,   Reveal, and Corrupt  oracle queries are sent by *A* in any order at will.
- *A* chooses a fresh session on which to be tested by sending a  Test  query to the fresh oracle associated with the test session at some point during *G*. This chosen test session must be fresh (in the sense of Definition 2). Depending on a randomly chosen bit *b*, *A* is given either the actual session key or a session key drawn randomly from the session key distribution.
- *A* continues making any  Send,  Reveal, and  Corrupt  oracle queries of its choice.
- *A* will eventually terminate *G* and outputs its guess of the value of *b*, denoted as *b*′.

We measure *A*'s success in *G* in terms of *A*'s advantage in distinguishing whether *A* receives the real key or a random value (i.e., whether *b*′ = *b*).

Let *k* be a security parameter. Then, the advantage function of *A* is denoted by  Adv^*A*^(*k*), where

(1) $$\begin{array}{c} Adv^{A}\left(k\right)=2\times Pr\left[b^{\prime }=b\right]-1. \end{array}$$

**Definition 3 (Definition of Security).**

A protocol is secure in the Bellare-Rogaway model if both the following requirements are satisfied.

1. Two oracles accept the same key when the protocol is run in the absence of a malicious adversary.
2. For all probabilistic, polynomial-time (PPT) adversaries  *A*, Adv^*A*^(*k*) is negligible.

## 3. A Provably-Secure SM2 Key Exchange Protocol

### 3.1. SM2 Key Exchange Protocol

The notations used in SM2 protocol (Table 1) are as follows:

- *A*, *B*: two SM2 protocol participants with identities ID_*A*_ and ID_*B*_ respectively,
- *a*, *b*: *a*, *b* ∈ *f*_*q*_: the parameters of the elliptic curve *E* on *F*_*q*_ where elliptic function is *y* ^2^ = *x* ^3^ + *ax* + *b*,
- *F*_*q*_: the prime field *F* includes *q* elements,
- *E*(*F*_*q*_): the set of all points on the elliptic curve *E* defined over *F*_*q*_,
- *G*: the base point of elliptic curve, order of *G* is prime number that *G* = (*x*_*G*_, *y*_*G*_),
- *h*: cofactor, *h* = #*E*(*F*_*q*_)/*n*, *n* is the order of *G*,
- *D*: the space of number that *D* = [1, *n* − 1],
- (*d*, *P*): long-term private and public key pair,
- *K*: session key,
- ID: identification of client,
- *Z*: hash value of identification, the length of *Z* is *entlen*_*A*_ bits,
- *H*_*v*_(): one-way hash function,
- (*r*, *R*): temporary private and public key pair,
- *klen* and *entlen*: the bit length of the key and ID, respectively,
- KDF(*Z*, *klen*): the one-way key derivation hash function whose output length is *klen*,
- *x*||*y*: concatenation of two strings *x* and *y*,
- ⊥: there is no message or the value is not known.

*A* randomly selects *d*_*A*_ ∈ [1, *n* − 1] and computes *P*_*A*_ = [*d*_*A*_]*G* = (*x*_*A*_, *y*_*A*_), prior to sending *P*_*A*_ to *B*. *B* will also randomly select *d*_*B*_ ∈ [1, *n* − 1] and compute *P*_*B*_ = [*d*_*B*_]*G* = (*x*_*B*_, *y*_*B*_), before sending *P*_*B*_ to *A*. The public parameters are (*E*(*Fq*), *G*, *n*, *Z*_*A*_, *Z*_*B*_, *P*_*A*_, *P*_*B*_). And *Z*_*A*_ and *Z*_*B*_ are hash values of the identification of *A* and *B*, respectively, where *Z*_*A*_ = *H*(*ENTL*_*A*_||ID_*A*_||*a*||*b*||*x*_*G*_||*y*_*G*_||*x*_*A*_||*y*_*A*_) and *Z*_*B*_ = *H*(*ENTL*_*B*_||ID_*B*_||*a*||*b*||*x*_*G*_||*y*_*G*_||*x*_*B*_||*y*_*B*_). To establish a session key with client *B*,

1. client *A* will now run the protocol as follows:
  - randomly selects *r*_*A*_ ∈ [1, *n* − 1],
  - computes *R*_*A*_ = [*r*_*A*_]*G* = (*x*_1_, *y*_1_) and $\bar{x_{1}}=2^{w}+(x_{1}\&(2^{w}-1))$ where *w* = ⌈(⌈log⁡_2_(*n*)⌉/2)⌉ − 1,
  - computes $t_{A}=(d_{A}+\bar{x_{1}}\cdot r_{A})\mathrm{mod}n$,
  - sends *R*_*A*_ to client *B*;
2. upon receiving the message *R*_*A*_ from client *A*, *B* will perform the following:
  - randomly selects *r*_*B*_ ∈ [1, *n* − 1],
  - computes *R*_*B*_ = [*r*_*B*_]*G* = (*x*_2_, *y*_2_), $\bar{x_{2}}=2^{w}+(x_{2}\&(2^{w}-1))$, *t*_*B*_ = $(d_{B}+\bar{x_{2}}\cdot r_{B})\mathrm{mod}n$, $V=[h\cdot t_{B}](P_{A}+[\bar{x_{1}}]R_{A})=(x_{V},y_{V})$ and *K*_*B*_ = KDF(*x*_*V*_||*y*_*V*_||*Z*_*A*_||*Z*_*B*_, *klen*),
  - (optional for key confirmation) computes *S*_*B*_ = *H*(0*x*02||*y*_*V*_||*H*(*x*_*V*_||*Z*_*A*_||*Z*_*B*_||*x*_1_||*y*_1_||*x*_2_||*y*_2_)),
  - Sends *R*_*B*_ to client *A* (optional for key confirmation, *B* will also send *S*_*B*_ to *A*);
3. upon receiving the messages, *R*_*B*_ (and *S*_*B*_), from *B*, client *A* will perform the following:
  - computes $\bar{x_{2}}=2^{w}+(x_{2}\&(2^{w}-1))$, *U* = $\left[h\cdot t_{A}](P_{B}+[\bar{x_{2}}]R_{B}\right)$ = (*x*_*U*_, *y*_*U*_) and *K*_*A*_ = KDF(*x*_*U*_||*y*_*U*_||*Z*_*A*_||*Z*_*B*_, *klen*),
  - (optional for key confirmation) computes *S*_1_ = *H*(0*x*02||*y*_*U*_||*H*(*x*_*U*_||*Z*_*A*_||*Z*_*B*_||*x*_1_||*y*_1_||*x*_2_||*y*_2_)),
  - verifies $S_{1}\overset{?}{=}S_{B}$, and if it returns true then *A* is assured that *B* actually has possession of the session key, otherwise, terminates the protocol run and outputs ⊥,
    - (optional for key confirmation, computes *S*_*A*_ = *H*(0*x*03||*y*_*U*_||*H*(*x*_*U*_||*Z*_*A*_||*Z*_*B*_||*x*_1_||*y*_1_||*x*_2_||*y*_2_))),
    - Sends *S*_*A*_ to client *B*;
4. (optional for key confirmation) upon receiving the message (*S*_*A*_) from client *A*, client *B* will perform the following:
  - computes *S*_2_ = *H*(0*x*03||*y*_*V*_||*H*(*x*_*V*_||*Z*_*A*_||*Z*_*B*_||*x*_1_||*y*_1_||*x*_2_||*y*_2_)),
  - verifies whether $S_{A}\overset{?}{=}S_{2}$,
  - if the verification returns wrong, then client *B* terminates the protocol run and outputs ⊥,
  - otherwise, client *B* is assured that *A* actually has possession of the session key;
5. session key established is *K*_*A*_ = *K*_*B*_,
6. SID is (*R*_*A*_||*R*_*B*_) or (*R*_*A*_||*R*_*B*_||*S*_*B*_||*S*_*A*_) in the case where key confirmation is required.

### 3.2. Security Proof

The security of the protocol—see Theorem 5—is based on the ECDLP assumption (see Definition 4) in the random oracle model.

**Definition 4 (ECDLP Assumption).**

The ECDLP problem is defined as follows:

(2) Instance:  E∖Fp, P,Q∈E(Fp)Output:  k∈N∗, k>1  such  that  Q≡kPmod⁡p.

If we can solve the discrete logarithm problem (DLP) [26], then we can also (immediately) solve the ECDLP problem.

**Theorem 5 .**

SM2 protocol is secure in the sense of Definition 3 when the underlying hash and key derivation schemes are modelled as random oracles and the elliptic curve discrete logarithm problem (ECDLP) assumption is satisfied in *E*(*F*_*q*_).

The soundness requirement is trivial to verify. We will now concentrate on proving the indistinguishability requirement.

In the usual tradition of reductionist proofs, we assume that there exists an adversary *A* against the protocol (i.e., *A* has a nonnegligible advantage, *η*(*k*), where *k* is the security parameter), and we then construct a solver *S* that makes use of *A* to solve the ECDLP problem. In other words, *S* will simulate the view of *A* by answering all Send, Reveal, Corrupt, and Test queries of *A*. *S* will start by randomly selecting two users, *I* and *J*, and a session number, *i*, as the test session. *S* will also manage two random oracles, *H* and KDF, in order to answer *A*'s queries. More specifically when the *H* oracle is queried, *S* will check whether the tuple is already in the *H*-list and output the stored response. Otherwise, *S* will respond with the appropriate output, *H*(⋯), and adds the tuple (*H*(⋯), *U*, *i*) to the *H*-list. *S* will answer KDF queries in the same manner.

- Send  (*Ω*_*I*_, *Ω*_*J*_, *j*, *m*) queries: for any well-formed  Send queries from *A*, *S* can trivially answer with the right output as the protocol specification demands. Specifically, *S* answers the query as follows.
  - If *Ω*_*I*_ = initiator and *Ω*_*J*_ = responder, then the *S* will output the message *m* = (*R*_*I*_, *Z*_*I*_, *Z*_*J*_, *P*_*I*_, *P*_*J*_).
  - Consider the case that *Ω*_*J*_ = initiator, *Ω*_*I*_ = responder, and message *m* = *R*_*I*_.
    - If *S* has rejected the message *m*, then *S* will respond with ⊥. Otherwise, *S* will verify whether *m* is the right format or not.
    - If *m* verifies correctly, then *S* will output messages (*R*_*J*_, *S*_*J*_) to *A*. Otherwise, *S* will abort the simulation and output ⊥.
  - Assume *Ω*_*I*_ = initiator, *Ω*_*J*_ = responder, and messages *m* = *R*_*J*_, *S*_*J*_.
    - If *S* has rejected the messages *m*, then *S* will respond with ⊥. Otherwise, *S* will verify whether *m* is the right format or not.
    - If *m* verifies correctly, then *S* will output messages *S*_*I*_ to *A*. Otherwise, *S* will abort the simulation and output ⊥.
- Reveal  (*Ω*, *j*) queries: if *Ω*_*j*_ = *I*_*i*_ or *Ω*_*j*_ = *J*_*i*_, then *S* will abort the simulation and fail. Otherwise this query can be answered with the right session key as long as *U*_*j*_ has accepted and neither *Ω* nor its partner has been corrupted. However, such a session will be rendered unfresh.
- Corrupt  (*Ω*) queries: this query can be easily answered as per the protocol specifications, unless *Ω* = *I* or *Ω* = *J*. In the latter scenario, *S* will abort the simulation and fail.
- Test  (*Ω*_1_, *Ω*_2_, *j*, *m*) queries: if Π_*U*_1_,*U*_2__^*j*^ ≠ Π_*I*,*J*_^*i*^, then *S* will abort the simulation and fail. Otherwise, *S* will check whether Π_*Ω*_1_,*Ω*_2__^*j*^ has accepted and that the session is fresh. If so, *A* will be given either the actual session key or a session key drawn randomly from the session key distribution, depending on the randomly chosen bit *b*.

For *A* to distinguish whether the value returned is the actual session key or a session key drawn randomly from the session key distribution, *A* has to determine the correct values of *U* = (*x*_*U*_, *y*_*U*_) or *V* = (*x*_*V*_, *y*_*V*_) to compute the session key (since *K*_*I*_ = KDF(*x*_*U*_||*y*_*U*_||*Z*_*A*_||*Z*_*B*_, *klen*) and *K*_*J*_ = KDF(*x*_*V*_||*y*_*V*_||*Z*_*A*_||*Z*_*B*_, *klen*)). For this to happen,

(i) *A* has to guess the long-term private key *d*_*I*_ and short-term private key *r*_*I*_ in order to compute *t*_*I*_ and hence, the session key *K*_*I*_. If *A* is able to successfully guess *d*_*I*_ and *r*_*I*_ via *P*_*I*_ = [*d*_*I*_]*G* and *R*_*I*_ = [*r*_*I*_]*G*, then *S* would be able to use *A* to solve the ECDLP problem. Let Succ_*d*_*I*__^*ECDLP*^ and Succ_*r*_*I*__^*ECDLP*^ denote *A*'s advantage in computing the correct values of *d*_*I*_ and *r*_*I*_, respectively, and Succ_*A*_^*U*^ denotes the event that *A* can successfully guess the session key using the computed values of *d*_*I*_ and *r*_*I*_. So we have
  (3) $$\begin{array}{c} \mathrm{Pr}\left[{\text{Succ}}_{d_{I}}^{ECDLP}\right]=\frac{1}{\left|D\right|};\;\;\mathrm{Pr}\left[{\text{Succ}}_{r_{I}}^{ECDLP}\right]=\frac{1}{\left|D\right|},\\ \mathrm{Pr}\left[{\text{Succ}}_{A}^{U}\right]=\mathrm{Pr}\left[{\text{Succ}}_{d_{I}}^{ECDLP}\right]\cdot \mathrm{Pr}\left[{\text{Succ}}_{r_{I}}^{ECDLP}\right]=\frac{1}{{\left|D\right|}^{2}}. \end{array}$$
(ii) *A* has to guess the correct value of *V*. Similar to the above, we let Succ_*d*_*J*__^ECDLP^ and Succ_*r*_*J*__^*ECDLP*^ denote *A*'s advantage in computing the correct value of *d*_*J*_ and *r*_*J*_, respectively, and Succ_*A*_^*V*^ denotes the event that *A* can successfully guess the session key using the computed values of *d*_*J*_ and *r*_*J*_. So we have
  (4) Pr⁡[SuccdJECDLP]=1|D|;  Pr⁡[SuccrJECDLP]=1|D|,Pr⁡[SuccAV]=Pr⁡[SuccdJECDLP]·Pr⁡[SuccrJECDLP]=1|D|2.

There is, therefore, a negligible advantage of *A* distinguishing whether the value returned is the actual session key or a session key drawn randomly from the session key distribution. Let *q*_*t*_ and Succ_*A*_^SM2^ denote the number of  Test  queries asked and the event that *A* can correctly distinguish the session key, respectively. We now have

(5) Pr⁡[SuccASM2]=(1−Pr⁡[SuccAU¯]Pr⁡[SuccAV¯])·qt=(1−(1−1|D|2)·(1−1|D|2))·qt=2qt|D|2−qt|D|4.

Since *D* = [1, *n* − 1], *n* → *∞*, *D* → *∞*, 1/|*D*|^2^ → 0, 1/|*D*|^4^ → 0, *q*_*t*_ ≪ *D*, and *q*_*t*_/|*D*|^2^ → 0, *q*_*t*_/|*D*|^4^ → 0, we have ((2*q*_*t*_/|*D*|^2^) − (*q*_*t*_/|*D*|^4^)) → 0 and ((2*q*_*t*_/|*D*|^2^) − (*q*_*t*_/|*D*|^4^)) = (*q*_*t*_/|*D*|^2^)(2 − (1/|*D*|^2^)) > 0. Therefore,

(6) $$\begin{array}{c} \mathrm{Pr}\left[{\text{Succ}}_{A}^{\text{SM}2}\right]=\frac{2q_{t}}{{\left|D\right|}^{2}}-\frac{q_{t}}{{\left|D\right|}^{4}}⟶0. \end{array}$$

This concludes the proof for Theorem 5.

## 4. A Provably-Secure Simplified SM2 Key Exchange Protocol

In this section, we propose a more efficient version of the SM2 protocol—see Table 2—and prove its security in the Bellare-Rogaway model.

### 4.1. Protocol Description

*A* randomly selects *d*_*A*_ ∈ [1, *n* − 1] and computes long-term public key *P*_*A*_ = [*d*_*A*_]*G* = (*x*_*A*_, *y*_*A*_) and *Z*_*A*_ = *H*(*ENTL*_*A*_||ID_*A*_||*a*||*b*||*x*_*G*_||*y*_*G*_||*x*_*A*_||*y*_*A*_). It then sends *P*_*A*_ and *Z*_*A*_ to *B*. *B* also randomly selects *d*_*B*_ ∈ [1, *n* − 1] and computes *P*_*B*_ = [*d*_*B*_]*G* = (*x*_*B*_, *y*_*B*_) and *Z*_*B*_ = *H*(*ENTL*_*A*_||ID_*A*_||*a*||*b*||*x*_*G*_||*y*_*G*_||*x*_*B*_||  *y*_*B*_), prior to sending *P*_*B*_ and *Z*_*B*_ to *A*. *E*(*Fq*),  *G*,  *n*,  *H*(),  KDF(),  *G*,  *P*_*A*_,  *P*_*B*_,  *Z*_*A*_,  *Z*_*B*_ are system parameters. To establish a session key with *B*,

1. *A* will now run the protocol as follows:
  - randomly selects *r*_*A*_ ∈ [1, *n* − 1],
  - computes *t*_*A*_ = (*d*_*A*_ · *r*_*A*_)mod⁡*n* and *R*_*A*_ = [*t*_*A*_]*G* = (*x*_1_, *y*_1_),
  - sends *R*_*A*_ to *B*;
2. upon receiving *P*_*A*_ from *A*, *B* will perform the following:
  - randomly selects *r*_*B*_ ∈ [1, *n* − 1],
  - computes *t*_*B*_ = (*d*_*B*_ · *r*_*B*_)mod⁡*n*, *R*_*B*_ = [*t*_*B*_]*G* = (*x*_2_, *y*_2_), *V* = [*t*_*B*_] · *R*_*A*_ + *d*_*B*_ · *P*_*A*_ = (*x*_*V*_, *y*_*V*_), and *K*_*B*_ = KDF(*x*_*V*_||*y*_*V*_||*Z*_*A*_||*Z*_*B*_, klen),
  - (optional for key confirmation) computes *S*_*B*_ = *H*(0*x*02||*y*_*V*_||*H*(*x*_*V*_||*Z*_*A*_||*Z*_*B*_||*x*_1_||*y*_1_||*x*_2_||*y*_2_)),
  - sends *R*_*B*_ to *A* (optionally for key confirmation, *B* will also send *S*_*B*_ to *A*);
3. upon receiving *R*_*B*_ (and *S*_*B*_, optionally for key confirmation) from *B*, *A* will perform the following:
  - computes *U* = [*t*_*A*_] · *R*_*B*_ + *d*_*A*_ · *P*_*B*_ = (*x*_*U*_, *y*_*U*_) and *K*_*A*_ = KDF(*x*_*U*_||*y*_*U*_||*Z*_*A*_||*Z*_*B*_, *klen*),
  - (optional for key confirmation) compute *S*_1_ = *H*(0*x*02||*y*_*U*_||*H*(*x*_*U*_||*Z*_*A*_||*Z*_*B*_||*x*_1_||*y*_1_||*x*_2_||*y*_2_)),
  - verifies that $S_{1}\overset{?}{=}S_{B}$, and if it returns true, then *A* is assured that *B* actually has possession of the session key, otherwise, terminates the protocol run and outputs ⊥,
    - (optional for key confirmation) computes *S*_*A*_ = *H*(0*x*03||*y*_*U*_||*H*(*x*_*U*_||*Z*_*A*_||*Z*_*B*_||*x*_1_||*y*_1_||*x*_2_||*y*_2_)),
    - Send *S*_*A*_ to client *B*;
4. (optional for key confirmation) upon receiving *S*_*A*_, *B* will perform the following:
  - computes *S*_2_ = *H*(0*x*03||*y*_*V*_||*H*(*x*_*V*_||*Z*_*A*_||*Z*_*B*_||*x*_1_||*y*_1_||*x*_2_||*y*_2_)),
  - verifies whether $S_{A}\overset{?}{=}S_{2}$,
  - if the verification returns wrong, then client *B* terminates the protocol run and outputs ⊥. If it returns true, then *B* is assured that *A* actually has possession of the session key. Otherwise, terminates the protocol run and outputs ⊥;
5. * *session key established is *K*_*A*_ = *K*_*B*_,
6. * *SID is (*R*_*A*_||*R*_*B*_) or (*R*_*A*_||*R*_*B*_||*S*_*B*_||*S*_*A*_) in the case where key confirmation is required.

### 4.2. Security Proof

**Theorem 6 .**

The simplified SM2 protocol (Table 2) is secure in the sense of Definition 3 when the underlying hash and key derivation schemes are modelled as random oracles and the ECDLP assumption is satisfied in *E*(*F*_*q*_).

The proof process is similar to that of Section 3.2.

- Send  (*Ω*_*I*_, *Ω*_*J*_, *j*, *m*) queries: for any well-formed Send queries from *A*, *S* can trivially answer with the right output as the protocol specification demands. Specifically, *S* answers the query as follows.
  - If *Ω*_*I*_ = initiator and *Ω*_*J*_ = responder, then the *S* will output the message, *m* = *R*_*I*_, to the query.
  - Consider the case that *Ω*_*J*_ = initiator, *Ω*_*I*_ = responder, and messages *m* = *R*_*I*_.
    - If *S* has rejected the message *m*, then *S* will respond with ⊥. Otherwise, *S* will verify whether *m* is the right format or not.
    - If *m* verifies correctly, then *S* will output messages (*R*_*J*_, *S*_*J*_) to *A*. Otherwise, *S* will abort the simulation and output ⊥.
  - Assume *Ω*_*I*_ = initiator, *Ω*_*J*_ = responder, and messages *m* = *R*_*J*_, *S*_*J*_.
    - If *S* has rejected the messages *m*, then *S* will respond with ⊥. Otherwise, *S* will verify whether *m* is the right format or not.
    - If *m* verifies correctly, then *S* will output messages *S*_*I*_ to *A*. Otherwise, *S* will abort the simulation and output ⊥.

Simulations for the Reveal, Corrupt, and Test follow that of Section 3.2.

For *A* to distinguish whether the value returned is the actual session key or a session key drawn randomly from the session key distribution (i.e., whether *b* = 0 or *b* = 1), *A* has to determine the correct values of *U* = (*x*_*U*_, *y*_*U*_) or *V* = (*x*_*V*_, *y*_*V*_) (since *K*_*I*_ = KDF(*x*_*U*_||*y*_*U*_||*Z*_*A*_||*Z*_*B*_, *klen*) and *K*_*J*_ = KDF(*x*_*V*_||*y*_*V*_||*Z*_*A*_||*Z*_*B*_, *klen*)). For this to happen, *A* has to obtain the correct value of *d*_*I*_ · *r*_*I*_ in order to compute *t*_*I*_ and consequently, the session key *K*_*I*_. For *A* to obtain *t*_*I*_, *A* has to be able to compute from *R*_*I*_ since *R*_*I*_ = [*t*_*I*_]*G*.

Let Succ_*t*_*I*__^*ECDLP*^ denote *A*'s advantage in computing *t*_*I*_ from *R*_*I*_, and we have

(7) $$\begin{array}{c} \mathrm{Pr}\left[{\text{Succ}}_{t_{I}}^{ECDLP}\right]=\frac{1}{\left|D\right|}. \end{array}$$

Let Succ_*t*_*J*__^*ECDLP*^ denote *A*'s advantage in computing *t*_*J*_ from *R*_*J*_, and we have

(8) $$\begin{array}{c} \mathrm{Pr}\left[{\text{Succ}}_{t_{J}}^{ECDLP}\right]=\frac{1}{\left|D\right|}. \end{array}$$

Let Succ_*A*_^ESM2^ denote the event that *A* is able to distinguish whether the value returned is the actual session key or a session key drawn randomly from the session key distribution. We then have

(9) Pr⁡[SuccAESM2]=(1−Pr⁡[SucctIECDLP¯]·Pr⁡[SucctJECDLP¯])·qt=(1−(1−1|D|)2)·qt=2qt|D|−qt|D|2.

Since  *D* = [1, *n* − 1], *n* → *∞*, *D* → *∞*, 2/|*D*| → 0, 1/|*D*|^4^ → 0, *q*_*t*_ ≪ *D*, and 2*q*_*t*_/|*D*| → 0, *q*_*t*_/|*D*|^2^ → 0, we have ((2*q*_*t*_/|*D*|)  −  (*q*_*t*_/|*D*|^2^)) → 0 and ((2*q*_*t*_/|*D*|) − (*q*_*t*_/|*D*|^2^)) = (*q*_*t*_/|*D*|)(2 − (1/|*D*|)) > 0. It follows that *Pr*⁡[Succ_*A*_^ESM2^] = (2*q*_*t*_/|*D*|) − (*q*_*t*_/|*D*|^2^) → 0.

This concludes the proof for Theorem 6.

## 5. Conclusion

Key exchange protocols are the cornerstone of any secure communication. By proving the widely used Chinese Government SM2 protocol secure in the Bellare-Rogaway model under the ECDLP assumption, we hope that this provides a strong assurance to protocol implementers that the protocol is behaving as desired. In addition, we presented a more efficient version of the SM2 protocol with a proof of security in the Bellare-Rogaway model under the ECDLP assumption.

A comparison with six existing pairing-free protocols reveals that the computational load of our simplified SM2 protocol is no more than that of the six and the SM2 protocols, yet provides both implicit key confirmation (*A* is assured that *B* can compute the session key) and explicit key confirmation (*A* is assured that *B* has actually computed the session key)—see Table 3. In Table 3, *a*, *m*, *e*, and *h* denote addition, multiplication, exponentiation, and hash operations, respectively.

## Figures and Tables

**Table 1:** SM2 key exchange protocol.

*A*	*B*
*d*_*A*_, *E*(*F*_*q*_), *G*, *n*, *Z*_*A*_, *Z*_*B*_, *P*_*A*_, *P*_*B*_, *H*(), KDF(), *G*	*d*_*B*_, *E*(*F*_*q*_), *G*, *n*, *Z*_*A*_, *Z*_*B*_, *P*_*A*_, *P*_*B*_, *H*(), KDF(), *G*
Randomly selects *r*_*A*_ ∈ [1, *n* − 1]	Randomly selects *r*_*B*_ ∈ [1, *n* − 1]
Computes *R*_*A*_ = [*r*_*A*_]*G* = (*x*_1_, *y*_1_)	Computes *R*_*B*_ = [*r*_*B*_]*G* = (*x*_2_, *y*_2_)
Computes $\bar{x_{1}}=2^{w}+(x_{1}\&(2^{w}-1))$	Computes $\bar{x_{2}}=2^{w}+(x_{2}\&(2^{w}-1))$
Computes $t_{A}=(d_{A}+\bar{x_{1}}\cdot r_{A})\mathrm{mod}n$	Computes $t_{B}=(d_{B}+\bar{x_{2}}\cdot r_{B})\mathrm{mod}n$
$\overset{R_{A}}{\to }$	
	Computes $\bar{x_{1}}=2^{w}+(x_{1}\&(2^{w}-1))$
	Computes $V=[h\cdot t_{B}](P_{A}+[\bar{x_{1}}]R_{A})=(x_{V},y_{V})$
	Computes *K*_*B*_ = KDF(*x*_*V*_||*y*_*V*_||*Z*_*A*_||*Z*_*B*_, klen)
	Computes *S*_*B*_ = *H*(0*x*02||*y*_*V*_||*H*(*x*_*V*_||*Z*_*A*_||*Z*_*B*_||*x*_1_||*y*_1_||*x*_2_||*y*_2_))
$\overset{R_{B},S_{B}}{\leftarrow }$	
Computes $\bar{x_{2}}=2^{w}+(x_{2}\&(2^{w}-1))$	
Computes $U=[h\cdot t_{A}](P_{B}+[\bar{x_{2}}]R_{B})=(x_{U},y_{U})$	
Computes *K*_*A*_ = KDF(*x*_*U*_||*y*_*U*_||*Z*_*A*_||*Z*_*B*_, klen)	
Computes *S*_1_ = *H*(0*x*02||*y*_*U*_||*H*(*x*_*U*_||*Z*_*A*_||*Z*_*B*_||*x*_1_||*y*_1_||*x*_2_||*y*_2_))	
Verifies $S_{1}\overset{?}{=}S_{B}$	
Computes *S*_*A*_ = *H*(0*x*03||*y*_*U*_||*H*(*x*_*U*_||*Z*_*A*_||*Z*_*B*_||*x*_1_||*y*_1_||*x*_2_||*y*_2_))	
$\overset{S_{A}}{\to }$	
	Computes *S*_2_ = *H*(0*x*03||*y*_*V*_||*H*(*x*_*V*_||*Z*_*A*_||*Z*_*B*_||*x*_1_||*y*_1_||*x*_2_||*y*_2_))
	Verifies $S_{2}\overset{?}{=}S_{A}$
Key established *K*_*A*_ = *K*_*B*_	Key established *K*_*B*_ = *K*_*A*_
SID is (*R*_*A*_||*R*_*B*_) or (*R*_*A*_||*R*_*B*_||*S*_*B*_||*S*_*A*_) in the case where key confirmation is required.	

**Table 2:** Simplified SM2 key exchange protocol.

*A*	*B*
pub: *E*(*F*_*q*_), *G*, *n*, *H*(), KDF(), *G*, *P*_*A*_, *P*_*B*_, *Z*_*A*_, *Z*_*B*_	
Randomly selects *r*_*A*_ ∈ [1, *n* − 1]	Randomly selects *r*_*B*_ ∈ [1, *n* − 1]
Computes *t*_*A*_ = (*d*_*A*_ · *r*_*A*_)mod⁡*n*	Computes *t*_*B*_ = (*d*_*B*_ · *r*_*B*_)mod⁡*n*
Computes *R*_*A*_ = [*t*_*A*_]*G* = (*x*_1_, *y*_1_)	Computes *R*_*B*_ = [*t*_*B*_]*G* = (*x*_2_, *y*_2_)
$\overset{R_{A}}{\to }$	
	Computes *V* = [*t*_*B*_] · *R*_*A*_ + *d*_*B*_ · *P*_*A*_ = (*x*_*V*_, *y*_*V*_)
	Computes *K*_*B*_ = KDF(*x*_*V*_||*y*_*V*_||*Z*_*A*_||*Z*_*B*_, klen)
	Computes *S*_*B*_ = *H*(0*x*02||*y*_*V*_||*H*(*x*_*V*_||*Z*_*A*_||*Z*_*B*_||*x*_1_||*y*_1_||*x*_2_||*y*_2_))
$\overset{R_{B},S_{B}}{\leftarrow }$	
Computes *U* = [*t*_*A*_] · *R*_*B*_ + *d*_*A*_ · *P*_*B*_ = (*x*_*U*_, *y*_*U*_)	
Computes *K*_*A*_ = KDF(*x*_*U*_||*y*_*U*_||*Z*_*A*_||*Z*_*B*_, klen)	
Computes *S*_1_ = *H*(0*x*02||*y*_*U*_||*H*(*x*_*U*_||*Z*_*A*_||*Z*_*B*_||*x*_1_||*y*_1_||*x*_2_||*y*_2_))	
Verifies $S_{1}\overset{?}{=}S_{B}$	
Computes *S*_*A*_ = *H*(0*x*03||*y*_*U*_||*H*(*x*_*U*_||*Z*_*A*_||*Z*_*B*_||*x*_1_||*y*_1_||*x*_2_||*y*_2_))	
$\overset{S_{A}}{\to }$	
	Computes *S*_2_ = *H*(0*x*03||*y*_*V*_||*H*(*x*_*V*_||*Z*_*A*_||*Z*_*B*_||*x*_1_||*y*_1_||*x*_2_||*y*_2_))
	Verifies $S_{2}\overset{?}{=}S_{A}$
Key established *K*_*A*_ = *K*_*B*_	Key established *K*_*B*_ = *K*_*A*_
SID is (*R*_*A*_||*R*_*B*_) or (*R*_*A*_||*R*_*B*_||*S*_*B*_||*S*_*A*_) in the case where key confirmation is required.	

**Table 3:** Protocol comparison.

Protocol	Cost	Both implicit and explicit key confirmation?
Cao et al. (2008) [13]	10*m* + 4*a* + 4*h* + 1*e*	No
Cao et al. (2010) [10]	8*m* + 3*a* + 2*h*	No
He et al. (2012) [2]	6m + 5a + 2e + 2h	No
Yang and Tan (2011) [24]	6*m* + 2*h*	No
He et al. (2011) [14]	5*m* + 4*a* + 2*h*	No
Chen and Han (2013) [25]	9*m* + 2*a* + 7*h* + 1*e*	No
SM2 [15]	5*m* + 4*a* + 3*h*	Yes
Our simplified SM2 protocol	4*m* + 1*a* + 3*h*	Yes
